# When to vaccinate for seasonal influenza? Check the peak forecast

**DOI:** 10.21203/rs.3.rs-4893237/v1

**Published:** 2024-09-09

**Authors:** Julie A. Spencer, Manhong Z. Smith, Dave Osthus, Prescott C. Alexander, Sara Y. Del Valle

**Affiliations:** 1* Information Systems and Modeling Group, Los Alamos National Laboratory, Bikini Atoll Rd., Los Alamos, 87545, NM, USA.; 2* Formerly Information Systems and Modeling Group, Los Alamos National Laboratory, Bikini Atoll Rd., Los Alamos, 87545, NM, USA.; 3 Currently Real World Data & Analytics Department, Boehringer Ingelheim Pharmaceuticals, Inc., Ridgebury Rd., Ridgefield, 06877, CT, USA.; 4 Statistical Sciences Group, Los Alamos National Laboratory, Bikini Atoll Rd., Los Alamos, 87545, NM, USA.; 5 Physics and Chemistry of Materials Group, Los Alamos National Laboratory, Bikini Atoll Rd., Los Alamos, 87545, NM, USA.

**Keywords:** influenza, vaccine, vaccine effectiveness (VE), VE waning, influenza forecasting

## Abstract

**Background:**

Seasonal influenza infects 5–20% of people every year in the United States, resulting in hospitalizations, deaths, and adverse economic impacts. To mitigate these impacts, influenza vaccines are developed and distributed annually; however, growing evidence suggests that vaccine effectiveness (VE) wanes over the course of a flu season. Delaying influenza vaccination for older adults has attracted attention as a potential public health strategy. However, given the uncertainties in seasonal peak, vaccine effectiveness, and waning rates, postponing vaccination could also lead to increased morbidity, motivating an evaluation of a range of potential scenarios.

**Methods:**

We systematically investigated a broad range of vaccination start times for five age groups under six combinations of initial effectiveness and waning rates, based on influenza cases and vaccine uptake data from 10 influenza seasons. We defined the most favorable vaccination schedule as the one that resulted in the greatest reduction in disease burden.

**Results:**

In scenarios with fast waning, all age groups benefit from delaying vaccination regardless of initial VE and peak timing. In scenarios with slower waning, results are mixed. For the ≥65 group, high initial VE and slow waning suggests that in early-peaking seasons, early vaccination most effectively reduces disease burden, while in late-peaking seasons delaying vaccination is most effective. For the ≥65 group in medium and low initial VE, and slow waning scenarios, delaying vaccination appears to prevent the greatest number of cases, regardless of whether the season peaks early or late.

**Conclusion:**

The most favorable vaccination schedule is sensitive to changes in initial VE, waning rate, and peak timing. Given estimates of these quantities from statistical and immunological models and observations, our methods can inform vaccination recommendations in order to most effectively reduce the annual disease burden caused by seasonal influenza. Specifically, accurate peak timing forecasts for the upcoming season have the potential to guide decisions on when to vaccinate.

## 1 Background

Seasonal influenza causes substantial health and economic burden in the United States each year. The Centers for Disease Control and Prevention (CDC) estimates that from 2010–2020, annual influenza epidemics have resulted in 9–41 million illnesses, 140,000–710,000 hospitalizations, and 12,000–52,000 deaths [1]. Seasonal vaccination has been the most effective strategy to reduce influenza transmission and mitigate its potential impacts [2]. The U.S. Advisory Committee on Immunization Practices (ACIP) publishes recommendations regarding the use of influenza vaccine every flu season. The ACIP has recommended influenza vaccination to be offered to everyone over 6 months of age by the end of October [3]. For certain populations, flu vaccines begin to be administered as early as July, when the vaccines first become available [4, 5]. As a result, approximately 30% of all adults in the US are vaccinated by the end of October [6].

Several recent studies have presented evidence suggesting that intra-seasonal waning of influenza vaccine effectiveness (VE) exists and is an epidemiologically important phenomenon [7–9]. VE waning is defined as the reduction of vaccine-induced immunity during an influenza season while the virus is still actively circulating. Rambhia and Rambhia [10] and Roy and MacDougall [11] summarized a series of recent studies regarding intra-seasonal waning of influenza VE [12–25]. These studies found some degree of VE waning, although the estimated degree varied substantially. These results suggest that early influenza vaccinations (e.g., during summer) may be suboptimal since protection may be diminished during peak months of influenza activity [10].

Postponing influenza vaccination has attracted attention in the flu research community as a potential public health strategy to counteract VE waning. A few recent studies have discussed the risks and benefits of delaying influenza vaccination [26–28]. Using linear VE waning functions for each season from 2007 to 2016 with 2009 excluded, Costantino et al. [26] studied the impact of the influenza vaccination timing change and reduced vaccine coverage on health outcomes for two age groups, < 65 and ≥ 65, in Australia. They found that delaying vaccination could have a net negative impact, if it results in missed vaccination. However, it is unclear how sensitive their results are to different VE waning functions. In contrast, Newall et al. [27] investigated the impact of delaying vaccination using two VE waning scenarios (both VE waning functions start at 50%, one wanes over 26 weeks, and the other wanes over 52 weeks) in older adults in the U.S. They found net benefits of delaying vaccination based on the 2010/11 to 2015/16 seasons, even if the vaccine coverage is lowered in some cases. Ferdinands et al. [28] selected a single influenza season, the 2012/2013 season, and evaluated the impact of potential vaccination timing change for older adults in the U.S. They showed that delaying vaccination until October could lead to negative outcomes (i.e., more influenza hospitalizations), if that strategy resulted in a > 14% reduction in the total number of vaccinated older adults compared with what would have otherwise been expected during that period (i.e., prior to October). Limitations of these studies indicated a need for further investigations: more complete age stratification, analyses spanning multiple seasons, and a broader range of VE assumptions under more realistic scenarios.

To address this need, we investigated a broad range of influenza vaccination schedules for five age groups, assessed how the recommendations change under different seasonal VE and waning scenarios, and quantified how influenza activity timing (early or late peaking season) impacts the schedule. Using empirical data and five age cohorts, we modeled the first week of influenza vaccination to begin such that the largest percentage of disease burden (i.e., influenza-like illness cases for people < 65 and influenza hospitalizations for people ≥ 65) can be prevented in the US for each age group. Then, we used these results to assess the possibility of an age-tiered vaccination schedule. Finally, we explored the impact of an early or late peaking season on various schedules under different VE scenarios.

## 2 Methods

We estimated the proportion of disease burden prevented at the state level for each age cohort and each influenza season from 2010/2011 to 2019/2020 under different vaccination schedules and VE scenarios. We assumed that the historic patterns of vaccination uptake (the proportion of people getting vaccinated) remained unchanged, and we evaluated schedules shifted to begin from 1 – 20 weeks earlier (i.e., advancing vaccination) or 1 – 20 weeks later (i.e., postponing vaccination) relative to historic uptake patterns for each state in each season. We defined the most favorable vaccination schedule as the one that resulted in the highest proportion of disease burden prevented.

### 2.1 Data Sources

To estimate the national influenza burden for each age cohort, we used six data sources: Influenza-like Illness (ILI) [29], laboratory-confirmed influenza hospitalizations (FluSurv-NET) [30], National Ambulatory Medical Care Survey (NAMCS) [31], Flu Near You (FNY) Survey results (see ILI Case Data Calibration, Additional File 1), vaccine coverage data from FluVaxView [32], and age-specific population estimates [33].

We assumed that vaccination timing, surveillance reports of ILI cases, and influenza hospitalizations represent the distribution of disease burden in each season. Due to low flu incidence during the summer, fewer providers report ILI data during this time. The Weekly U.S. Influenza Summary Update used by CDC to monitor influenza activity is updated each week from October through May of each year [34]. Thus, we limited our analysis to ILI data from surveillance week 40 (first week of October) to surveillance week 22 (end of May or beginning of June) to represent the disease burden distribution for the flu season as framed by the CDC.

We further assumed (1) that the Flu Near You (FNY) survey respondents (see ILI Case Data Calibration, Additional File 1) constitute a representative sample of the population of each U.S. Census Region (note that this assumption–which is likely incorrect–will be addressed through a sensitivity analysis in Section 2.4); (2) that the data summarized from four seasons from 2015 to 2019 can be considered reasonable time-invariant approximations that may be applied to the ten seasons from 2010 to 2020, and (3) that the ratio of FNY symptom reports consistent with ILI to total FNY symptom reports equals the ratio of survey participants with ILI to total survey participants.

Based on ILINet’s reporting standards [29], we partitioned the population into five age cohorts: 0–4 years, 5–24 years, 25–49 years, 50–64 years, and ≥ 65 years (Table 1). For disease burden data, we used weekly ILI data from the U.S. Outpatient Influenza-like Illness Surveillance Network (ILINet) for people < 65 years [29] and laboratory-confirmed influenza hospitalizations (FluSurv-NET) for people ≥ 65 years [30]. We used two different types of data because ILI outpatient visits better reflect disease burden in people < 65, whereas hospitalizations better represent disease burden for people ≥ 65. Hospitalizations for a particular virus more accurately represent disease burden than do syndromic ILI case counts, which underestimate burden specifically for older adults [35, 36]. Note that this assumption is consistent with previously published studies such as Ferdinands et al. [28].

In the ILINet system, ILI is defined as a fever (temperature of 100°F [37.8°C] or greater) and a cough and/or a sore throat [37]. ILINet includes participating outpatient healthcare providers in all U.S. states, Puerto Rico, the District of Columbia and the U.S. Virgin Islands that report the total number of patients seen for any reason and the number of those patients with ILI by age group each week. All states were included in this analysis except Florida, as ILINet data are not publicly available for this state. Unlike ILINet, FluSurv-NET only covers selected states from season to season. We define a data point as a state in a particular season. Taking into account 49 states and 10 seasons, there are 116 data points for people from FluSurv-Net for the age group of ≥ 65 years and 490 data points from ILINet for each of the under 65 age groups.

**Fig. 1:**
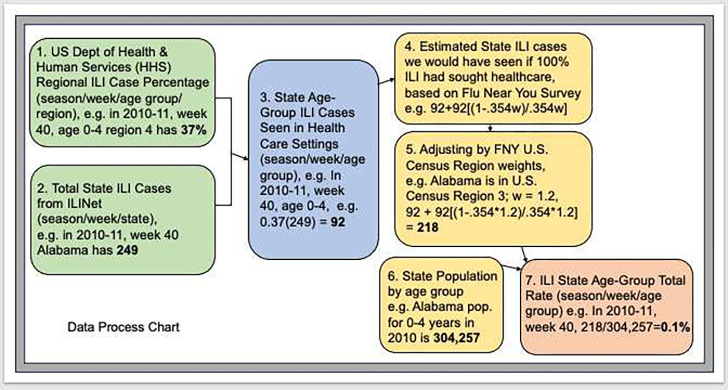
Process chart depicting data used to calculate state level total ILI cases by age cohort, using 0–4 age group and the state of Alabama as an example

For people aged ≥ 65 years, weekly rate of influenza hospitalizations is available from FluSurv-NET [30]. To calculate total hospitalizations, we multiplied the hospitalization rate by the total population of age ≥ 65 years at the state level. However, ILI case rates by age cohort at the state level are not available. ILINet data are only reported as total ILI cases from all patients seen by ILINet participating providers for any reason. Further, these data only represent people seen in healthcare settings and not the whole population. We accounted for these data constraints by calculating ILI case rate by age cohort at the state level as shown in Figure 1:

1. We calculated weekly cases by age cohort for each state (steps 1–3 in Figure 1). We used ILINet data at the Health and Human Services (HHS) region level, which is provided by age cohort. We applied the HHS ILI percentages for each cohort to the state total ILI cases to get state level ILI cases seen in healthcare settings by age cohort in each season. In doing so, we assumed that the HHS region level ILI prevalence represents the state level ILI prevalence among different age cohorts.
2. To estimate total prevalence, we used the results of the FNY survey to calculate the weekly state level ILI cases we would have seen if 100% of people experiencing ILI had sought health care (step 4 in Figure 1), and adjusted the state level ILI prevalence accordingly (step 5 in Figure 1). See ILI Case Data Calibration, Additional File 1, for details.
3. We multiplied the state case rate by state population for each age cohort to get total state ILI case rate (steps 6 and 7 in Figure 1).

### 2.2 Vaccine Coverage and Seasonal Influenza Peaks

In addition to seasonal VE, influenza vaccine uptake and the timing of influenza activity are the other two major factors that impact the outcome of our model. We used monthly data by age group and state from FluVaxView for vaccine coverage data [32]. See Monthly Coverage Data Preparation, Additional File 1, for our treatment of missing data problems. We then interpolated monthly coverage to estimate weekly coverage as follows. We assumed that vaccination begins at the end of July (surveillance week 30), and we assigned all vaccine coverage data for July (we assumed zero if not reported) to surveillance week 30. Cumulative monthly coverage data from surveillance week 30 to surveillance week 22 (end of May) of the following year were interpolated by fitting monotonic cubic splines to estimate weekly coverage. We assumed that a full immune response is achieved two weeks after vaccination, thus a vaccine received on surveillance week 30 becomes fully effective on surveillance week 32.

**Fig. 2:**
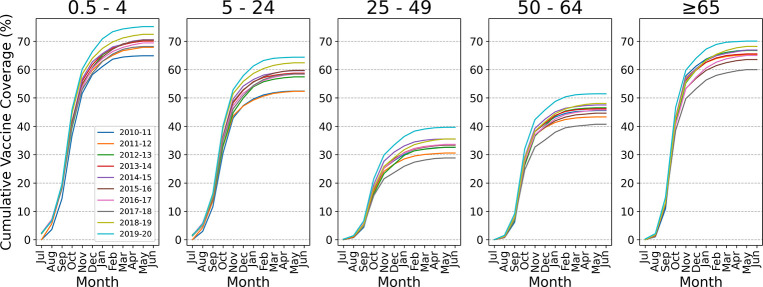
Cumulative nationwide monthly coverage by age group for seasons 2010/2011 to 2019/2020

We adopted the age cohorts from ILINet as the standard for our age cohorts; Table 1 shows the age cohort structure for the coverage, NAMCS, and FNY data used in this study. Vaccine coverage data is reported for the age groups of 6 months to 4 years and 6 months to 17 years. We derived coverage for the age group of 5 to 17 years from the difference of the above two age groups using coverage and population data. The vaccine uptake pattern and total vaccine coverage (Figure 2) showed substantial variation across age groups and seasons (see Note on Data Consistency, Additional File 1). Compared to the other three age groups, the youngest and the oldest age groups have much higher total coverage and faster vaccination uptake from July to November, at which point for all groups, coverage typically starts to flatten out. This uptake is represented by the slope of the cumulative coverage between July and November.

**Fig. 3:**
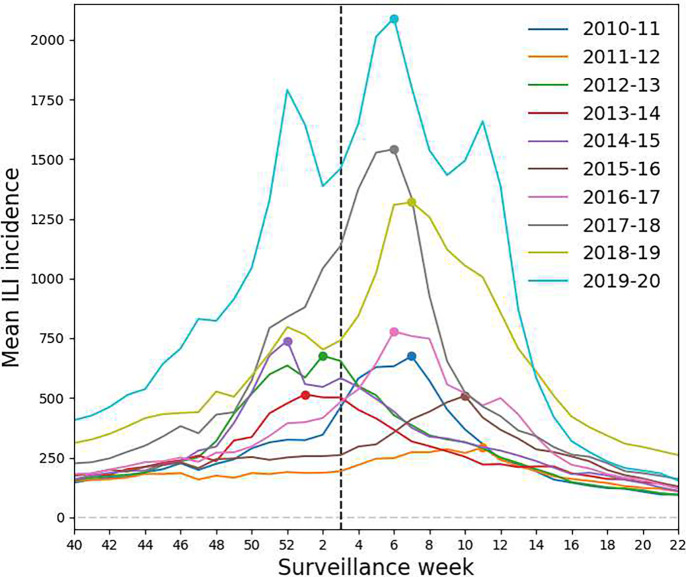
Mean incidence of influenza-like illness for ten seasons, 2010–2020. Influenza surveillance weeks from approximately October to May are shown on the x-axis; mean incidence is shown on the y-axis. Color indicates season. The dotted vertical line at Week 3 marks the partition between early and late peaking seasons, and the points on the curves indicate peak weeks.

To assess the potential impact of influenza peak timing, we separated the results for states that experienced early peaking (peak week at or before the third week of January) from those for states that had late peaking (peak week after the third week of January) across all ten seasons. Based on historical data, influenza activity typically peaks between December and February [34]; however, a flu season can have multiple peaks. We defined the peak week for each season as the week at which the largest number of ILI cases are observed. When more than one equivalent week existed, we chose the first one. As shown in Figure 3, historical influenza peak weeks at the state level varied among seasons.

### 2.3 Model

We simulated six scenarios for each age group. Each scenario uses a different VE waning function and has either 490 (< 65 years) or 116 (≥ 65 years) data points corresponding to a total of 10 seasons. Each data point is defined as one state in a specific season for that age group (for example, Alabama in the 2010/2011 season for age group 0 – 4 years old). For each data point, we calculated the percentage of disease burden averted under the actual vaccination schedule and 40 shifted schedules (i.e., beginning from one to 20 weeks earlier or later than the actual schedule). Although the vaccination schedule is unlikely to be shifted by five months earlier due to various factors such as vaccine production and logistics, we included this wide range to ensure that the best shift would fall within the explored range. We calculated the percentage of disease burden prevented under the current and the shifted schedules for the season (i.e., surveillance week 40 to week 22 of the following year in our study). We defined the most favorable vaccination start week, that is, the optimal vaccination strategy, as the one resulting in the the greatest estimated reduction in disease burden.

Following Newall et al. [27] with revised notations, the proportion of prevented cases from vaccination ($p_{t}$) for each week is estimated as

(1) $$p_{t}=\sum_{s=0}^{t-2}c_{\left(t-2-s\right)}e_{s}$$

where t is time in weeks and s represents the number of weeks that have elapsed since the development of a full immune response (i.e., if it has been t weeks since the vaccine was administered, s=t-2). Note that we restrict t≥2 to account for the two-week lag of immune response development (we assume people are fully vaccinated two weeks after receiving the shot).

$\sum_{s=0}^{t-2}\;c_{\left(t-2-s\right)}$ is the cumulative vaccine coverage at week t while accounting for the two-week delay in the development of an immune response. Figure 2 shows cumulative vaccine coverage $c_{t}$, without the two-week delay. $e_{s}$ is the value of the vaccine efficacy function (i.e., VE waning function) on week s, and it ranges $[0,1].e_{0}$ is the initial VE value, where $e_{s}=0$ indicates that the vaccine is not protective and $e_{s}=1$ indicates that the vaccine is 100% effective.

For example, if the cumulative vaccine coverage is 40% on a given week of the influenza season for a given age group and $e_{s}=1,\;40\%$ of cases will have been averted, whereas (in a more realistic situation), if cumulative vaccine coverage remains the same and $e_{s}=0.5,\;20\%$ of cases will have been averted.

To calculate the baseline disease burden without vaccine at week $t,D_{t}^{\prime }$, we removed the vaccine effect from the reported disease burden, $D_{t}$, as follows,

(2) $$D_{t}^{\prime }=\frac{D_{t}}{1\;-\;p_{t}}$$

Then seasonal disease burden without vaccine, from surveillance week 40 to week 22 of the following year in our analysis, is represented by $\sum_{t}D_{t}^{\prime }$. Thus the seasonal percentage of disease burden prevented is represented by

(3) $$\frac{\sum_{t}D_{t}^{\prime }-\sum_{t}D_{t}}{\sum_{t}D_{t}^{\prime }}$$

**Fig. 4:**
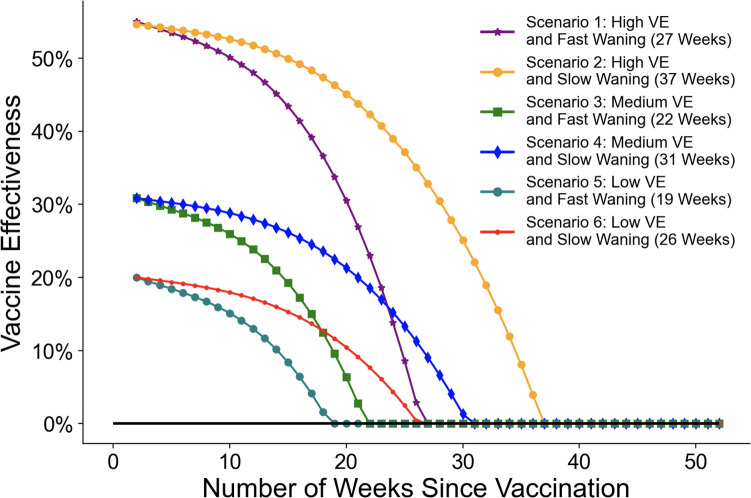
Vaccine effectiveness waning scenarios. The initial vaccine effectiveness is set two weeks after vaccination, when the waning process begins.

We considered six hypothetical vaccine effectiveness scenarios as shown in Figure 4 to account for three levels of starting VE (high, medium, and low) and two effectiveness waning functions (fast and slow). Note that VE and waning are assumed to be the same across all age groups and that we constrain negative values of VE to zero, although a value of 0 for VE is likely a pessimistic assumption.

#### 2.3.1 High VE and Fast Waning

For the first scenario, we adapted the waning model fitted to empirical data by Ferdinands et al. [28]. We modified their original equation by starting waning two weeks after vaccination, and by changing the time unit to one week instead of bi-week for t. This scenario assumes a high VE of 55% and fast waning over 27 weeks with a season average waning rate of approximately 7%.


(4) $$VE_{1}=max\left(0,\;55-1.37t+0.18t^{2}-0.03t^{3}\right)$$

#### 2.3.2 High VE and Slow Waning (Best Scenario)

In the second scenario, we assume the same high initial VE as the first scenario and slower waning, dropping to zero after 37 weeks.


(5) $$VE_{2}=max\left(0,\;55-0.5t+0.05t^{2}-0.01t^{3}\right)$$

#### 2.3.3 Medium VE and Fast Waning

We also adapted the third scenario from Ferdinands et al., which was fitted to empirical data [28]. It has an initial VE of 30.85% and wanes quickly, over 22 weeks.


(6) $$VE_{3}=max\left(0,\;30.85-1.37t+0.18t^{2}-0.03t^{3}\right)$$

#### 2.3.4 Medium VE and Slow Waning

In the fourth scenario, we assume the same initial medium VE as in the third scenario, with slower waning of 31 weeks.


(7) $$VE_{4}=max\left(0,\;30.85-0.5t+0.05t^{2}-0.01t^{3}\right)$$

#### 2.3.5 Low VE and Fast Waning (Worst Scenario)

The fifth scenario has an initial VE of 20% and wanes over 19 weeks.


(8) $$VE_{5}=max\left(0,\;20-1.37t+0.18t^{2}-0.03t^{3}\right)$$

#### 2.3.6 Low VE and Relatively Slow Waning

The sixth scenario has an initial VE of 20% and wanes over 26 weeks.


(9) $$VE_{6}=max\left(0,20-0.5t+0.05t^{2}-0.01t^{3}\right)$$

### 2.4 Sensitivity Analysis

Given the existence of uncertainty in weekly influenza case count estimates for ten seasons from 2010/2011 to 2019/2020, we conducted a sensitivity analysis to evaluate the robustness of our results with respect to the precise case estimation method used. For people < 65, we defined a lower bound (extreme underestimation) as the number of cases seen in health care settings divided by the entire population. In the underestimation, we are assuming that ILI cases seen in health care settings represent all cases that occurred. We defined an upper bound (extreme overestimation) by assuming that the proportion of ILI to people seen in health care settings equals the proportion of ILI in the entire population. These lower and upper bounds on the case count estimates take into account that the FNY survey may not be a representative sample of the underlying populations and may under or over estimate healthcare seeking behavior. The lower bound takes into account that ILI is likely an overestimate of influenza, since many viruses contribute to ILI. The sensitivity analysis is not applicable to people ≥ 65, as FluSurv-NET provides laboratory-confirmed influenza case counts for that age group.

## 3 Results

**Fig. 5:**
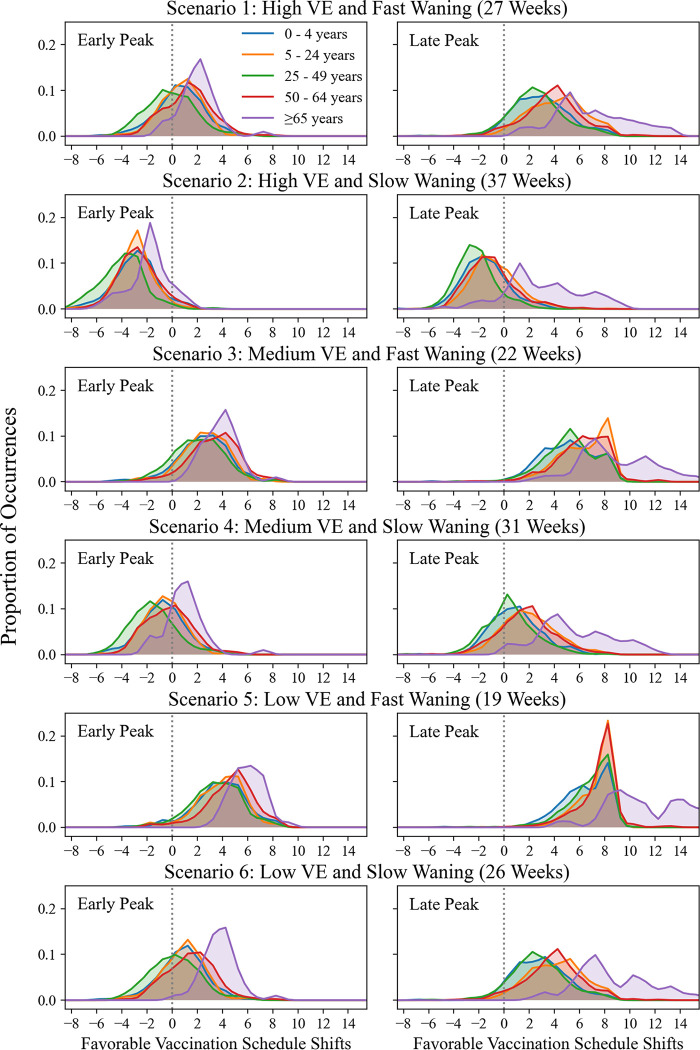
Distributions of favorable vaccination schedule shifts compared to historic uptake, under six different initial VE and waning scenarios. Data points are states/seasons. On the x-axes, zero indicates the starting historic vaccination uptake week for each state and season. Negative numbers indicate17 early vaccination in weeks; positive numbers indicate delayed vaccination in weeks. The y-axes show the proportion of occurrences of a particular schedule shift for all states and seasons in our data set. For visualization purposes, shift distributions are smoothed using a Gaussian with 0.5 week standard deviation.

In general, when VE waning is fast, delaying vaccination is beneficial in most states and seasons for most age groups. When waning is slow, results are mixed. The distributions of favorable schedule shifts for starting vaccination are shown in Figure 5 and the mean favorable shifts are shown in Table 2. See Additional File 2 for individual histograms for each age group.

In Scenario 1, for the case of late peaking seasons for ≥ 65, the average favorable shift indicates postponing vaccination by roughly seven weeks (Table 2). The absolute shift size is less than 4 weeks for all other age groups. In early peaking seasons, the best shift is postponing vaccination by about 2 weeks for ≥ 65, and absolute shift size is less than or equal to one week for all other age groups.

In Scenario 2, the schedule that prevents the most cases in the majority of the circumstances has vaccination starting earlier than historic uptake for all age groups with the exception of ≥ 65 for late peaking seasons (Figure 5). On average, the best shift for all age groups is to begin vaccination earlier by 3 to 4 weeks in the case of early peaking seasons, and by about one to two weeks in late peaking seasons, with the exception of the ≥ 65 group (Table 2). For ≥ 65, the average beneficial shift is to postpone vaccination by about three weeks for late peaking seasons.

Scenario 3 has the same waning rate (7% average monthly waning over 22 weeks) as does Scenario 1, but has a lower initial VE of 30.85%. In this scenario, the schedule shift that reduces the most burden for early and late peaking seasons and all age groups is to postpone vaccination.

In Scenario 3 for early peaking seasons, the average best shift for age groups 0–4 years, 5–24 years, and 25–49 years is to postpone vaccination by about one to two weeks, and for ≥ 65 by about three weeks. In the case of late peaking seasons, the average best shift for age groups < 65 is to postpone vaccination by about five to six weeks, and for ≥ 65 by nine weeks.

In Scenario 4, with initial VE of 30.85% and waning of 31 weeks, the schedule that begins about one to two weeks earlier than historic uptake averts the most cases in early peaking seasons, with the exception of ≥ 65, for which the best schedule is postponed by about half a week. The schedule that begins about one to six weeks later than historic uptake averts the most cases for late peaking seasons.

In both Scenarios 5 and 6, the initial VE is 20%. Scenario 5 has the shortest duration of protection at 19 weeks (i.e., it is the worst case among the six scenarios analyzed). In both scenarios, postponing vaccination reduces influenza burden for most states and seasons in all age groups, with the exception of the 25–49 group in Scenario 6, which benefits from starting vaccination at approximately the historic uptake point in the early peak case.

For each age group under all scenarios, there are differences in the average favorable shift when comparing late peaking seasons to early peaking seasons. In general, late peaking seasons suggest delaying vaccination, especially under the fast-waning scenarios. It should also be noted that the distribution of schedules is wider in the case of late peaking seasons because the window of early peaking (as early as mid-November to mid-January) is shorter than that of late peaking (mid-January to as late as mid-April). Additionally, we observe that for the ≥ 65 age cohort, postponing vaccination appears to be the most favorable recommendation across all scenarios, except for Scenario 2, early peak.

### 3.1 Sensitivity Analysis Results

Our sensitivity analysis shows that model outputs (distributions and means of favorable schedule shifts) for all scenarios are robust to variation in influenza case count estimates (see Additional File 3). Slight variation in the results exists for the overestimation, which is based on the assumption that the proportion of ILI cases seen in health care settings equals the proportion of ILI cases in the entire population. However, 100% of the 24 mean favorable schedule shifts resulting from this upper bound of ILI case estimates are within one week of those from the main analysis. Two out of these 24 mean schedule shifts differs in sign from those of the main analysis. As expected, the mean schedule shifts of the lower bound of ILI case estimates, which are a linear transformation of the main case estimates, are identical to those of the main case. Overall, despite a wide range of variation in the absolute numbers of estimated cases, the resulting optimal vaccination strategy remains the same. See Additional File 3 for table and plots.

## 4 Discussion

An ideal way to reduce influenza burden would be by means of a highly effective vaccination program that provides protection for the whole season. However, vaccine effectiveness varies and wanes. In this study, we quantified the impact of different vaccine effectiveness and waning scenarios based on 10 seasons of influenza case and vaccine uptake data to determine vaccination roll-out schedules that avert the greatest number of cases. Strengths of our study are stratification by five age categories, analyses of a broad spectrum of waning scenarios, and consideration of 40 vaccination start weeks, 1–20 before and 1–20 after the historic start week.

Our results show that it may be worthwhile to consider delaying vaccination if the waning rate is fast; yet if the VE wanes slowly, it is more challenging to determine an ideal influenza vaccination schedule. Additionally, our findings show that starting vaccination at the same time for all age groups may not be optimal. Nevertheless, our analysis supports current ACIP timing of vaccination recommendations that children can get their vaccine earlier in situations when initial VE is high and waning is slow, but that adults should avoid earlier vaccinations in most scenarios [5]. Our results indicate that postponing vaccination is favorable in most circumstances for the ≥ 65 group, with the exception of Scenario 2 (high VE and slow waning in early-peaking seasons). Thus, a general conclusion cannot be drawn in the absence of improved ability to predict the peak of the flu season. A tiered vaccination strategy can be implemented under the existing schedule to improve outcomes. Nonetheless, the decision of postponing or advancing vaccination for each age group cannot be determined without knowing how the vaccination coverage may potentially change, how the VE will wane, and when the influenza season will peak.

A challenge to consider is our limited understanding of VE waning and how to slow the waning of VE. Recently, many studies have evaluated how immunity wanes after vaccination [7–28]. However, results are largely inconsistent in terms of the estimated VE and the degree of waning. Better estimates of VE and its waning rate, as well as the data needed to support this, will be important in the future. To slow the waning, Rambhia and Rambhia (2018) recommended a mid-flu-season booster vaccine, and vaccine adjuvants or use of high-dose vaccines for susceptible populations. As of June 30, 2022, the CDC adopted ACIP’s recommendation that people > 65 be preferentially given high dose or adjuvanted flu vaccines [38]. However, limited studies on the outcomes of this recommendation have shown mixed results [39]. It remains to be seen how VE waning is impacted.

Finally, this work shows that accurate peak timing forecasts for flu seasons with actionable lead times can play an important role in vaccination start time decisions. Accurate and reliable long-lead peak time forecasts could guide public influenza vaccination campaigning efforts. In particular, seasonal forecasts can guide when people should start getting vaccinated (e.g., before Halloween). Such accurate peak time forecasts at this lead time do not currently exist (e.g., accurate early/late peak forecasts made in July), but infectious disease forecasting is an active area of research with real-time forecasting [40–42]. Additionally, new initiatives by the CDC Center for Forecasting and Outbreak Analytics [43], are promising in making these forecasts a reality. This work provides a practical and concrete example in which reliable forecasting efforts could help reduce the burden of seasonal influenza through annual fine tuning of vaccination start dates.

Note that our study did not specifically assess the impact of vaccination schedule changes on missed vaccination when the schedule is postponed. Rather, we focused on the fundamental issue of whether or not we have, or could acquire, enough information to change the existing vaccination schedule. However, our analysis does implicitly account for missed vaccinations due to shifts in vaccination start dates. Based on our assumption that the vaccine uptake pattern persisted under the shifted schedule, some portion of the population who were normally vaccinated late in the season would be shifted out of the postponed schedule in certain cases, because they effectively missed vaccination for that season. The impact of this issue, however, is minor relative to the large and important uncertainty in the estimate of the VE waning function itself.

There are several limitations to our study. First, we applied the same VE waning functions across seasons, while VE dynamics could differ from season to season depending on the circulating influenza virus strains and the match between the viruses and the vaccine [44–46]. Second, we did not specifically quantify the impact of precise influenza season peak timing on vaccination schedule; we only considered whether a season peaked early or late (see Methods, Section 2). Third, we did not explore potential differences in VE waning when history of repeated vaccinations is taken into account [47, 48]. Our VE waning functions are based on existing literature but additional studies are needed to more accurately quantify vaccine effectiveness and waning. Fourth, the VE functions assumed similar dynamics for all age groups and it is possible that each age group may have different dynamics [49]. Fifth, our models assume that shifting vaccination timing does not change people’s vaccination behavior (i.e., a shift in time does not change the shape of the vaccine coverage curves). While this assumption is unlikely to hold to high precision, by including data from 49 states and 10 seasons our results are unlikely to be appreciably affected by such changes in behavior (see Additional File 1 for further discussion). Finally, we used ILI data for people < 65 years of age, which includes non-influenza viral causes and as such, it is likely to overestimate influenza burden and increase uncertainty in its temporal dynamics [50]. Nonetheless, according to our sensitivity analysis, the results of our study are robust to wide variations in ILI case estimates, and the overall insights are unchanged.

## 5 Conclusions

Our results are consistent with current CDC influenza recommendations for vaccine timing, which are intended to guide influenza vaccination campaigns. However, given the uncertainty in VE effectiveness and waning rates, it may be beneficial to offer an annual mid-season booster vaccination to people ≥ 65 in order to reduce both morbidity and mortality in this population. Additionally, our results show that one size does not fit all, and that a tiered vaccination strategy may lead to more favorable outcomes.

## Figures and Tables

**Table 1:** Age group correspondences in years from different data sources for people < 65 years old

Historic Case Burden and Patient Data (ILINet)^1^	Vaccine Coverage Data (FluVaxView)	National Ambulatory Medical Care Survey (NAMCS)	Flu Near You (FNY) Survey

0 – 4	0.5 – 4	0 – 4	0 – 17
5 – 24	5 – 17	5 – 24	0 – 17
25 – 49	18 – 49	25 – 44	18 – 49
50 – 64	50 – 64	45 – 64	50 – 64

1 Used as the standard for this study.

**Table 2:** Mean shift in weeks that averts maximum cases, aggregated by age group and early or late peaking seasons under six VE and waning scenarios. Negative numbers indicate beginning vaccination sooner than historic uptake; positive numbers indicate beginning vaccination later than historic uptake.

Scenario	0–4 Early	0–4 Late	5–24 Early	5–24 Late	25–49 Early	25–49 Late	50–64 Early	50–64 Late	≥ 65 Early	≥ 65 Late
1	0.40	2.69	0.50	3.94	−0.32	2.36	1.04	3.64	1.82	7.10
2	−3.12	−1.76	−3.19	−0.98	−4.23	−2.45	−2.97	−1.29	−2.07	3.22
3	2.24	4.84	2.37	6.04	1.85	4.99	3.10	5.95	3.63	8.99
4	−0.96	0.74	−0.90	1.75	−1.85	0.27	−0.51	1.52	0.59	5.69
5	3.56	6.20	3.62	7.04	3.28	6.53	4.35	7.19	4.70	10.31
6	0.57	2.64	0.66	3.89	0.03	2.50	1.28	3.71	2.13	7.27

## Data Availability

All data are publicly available and fully cited.

## List of abbreviations

ACIP: US Advisory Committee on Immunization Practices
CDC: Centers for Disease Prevention and Control
HHS: Health and Human Services
ILINet: Influenza-like Illness Surveillance Network
NAMCS: National Ambulatory Medical Care Survey
VE: Vaccine Effectiveness
WHO: World Health Organization
